# Biomechanical evaluation of temporary epiphysiodesis at the femoral epiphysis using established devices from clinical practice

**DOI:** 10.1007/s10856-021-06515-9

**Published:** 2021-04-01

**Authors:** Charlotte Struwe, Sebastian G. Walter, Claudia Druschel, Rahel Bornemann, Milena Ploeger, Sebastian Koob, Richard Placzek

**Affiliations:** 1 Department for Orthopedic Surgery and Traumatology, Rheinische-Friedrich-Wilhelms-Universität, Bonn, Germany; 2 Department for Orthopedic Surgery and Traumatology, Carl-Gustav-Carus-Universität, Dresden, Germany

## Abstract

The aim of this study is to compare biomechanical features of different devices used in clinical routine for temporary epiphysiodesis (eight-Plate® and FlexTack^TM^). The tested implants were divided into four different groups (eight-Plate® vs. FlexTack^TM^ for lateral and anterior implantation) á 10 samples for testing implanted eight-Plate® vs. FlexTack^TM^ in fresh frozen pig femora for maximum load forces (*F*_max_) and axial physis distance until implant failure (*l*_max_). A servo hydraulic testing machine (858 Mini Bionix 2) was used to exert and measure reproducible forces. Statistical analyses tested for normal distribution and significant (*p* < 0.05) differences in primary outcome parameters. There were no significant differences between the eight-Plate® lateral group and the FlexTack^TM^ lateral group for neither *F*_max_ (*p* = 0.46) nor *l*_max_ (*p* = 0.65). There was a significant higher *F*_max_ (*p* < 0.001) and *l*_max_ (*p* = 0.001) measured in the eight-Plate® group compared to the FlexTack^TM^ group when implanted anteriorly. In anterior temporary ephiphysiodesis, eight-Plate® demonstrated superior biomechanical stability. At this stage of research, there is no clear advantage of either implant and the choice remains within the individual preference of the surgeon.

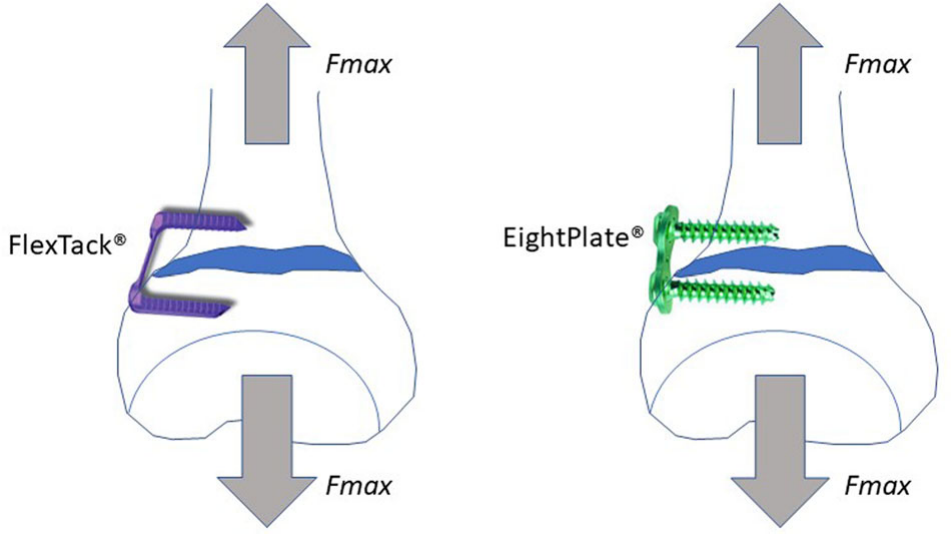

## Introduction

Angular deformities of the lower extremity—especially of the knee in the frontal plane—are considered as pre-arthrotic deformities [1, 2].

Treatment options to address these deformities are osteotomies that are surgically challenging and accompanied by a high risk of developing complications such as a compartment syndrome, neurovascular injuries, or over-/undercorrections [3]. Another disadvantage of osteotomies is the necessity of an internal or external fixation and thus limited weight bearing and mobilisation for at least 6 weeks after operation [3]. In case of open growth plates (in children and adolescents), corrective osteotomies of the distal femur or the proximal tibia can be avoided by growth guiding techniques such as (hemi)epiphysiodeses.

There are two categories of epiphysiodesis. The permanent and irreversible epiphysiodesis—originally described by Phemister—requires the destruction of the epiphyseal plate and is therefore indicated only when precisely calculating the remaining growth and determining the perfect age for surgery [3]. Canale and Christian [4] and Ogilvie and King [5] have focused on permanent percutaneous methods of minimal invasive epiphysiodesis using image intensification. Using these techniques, the physis is ablated or destroyed by drilling or curetting through small medial and lateral incisions. In 1998, Metaizeau et al. [6] described a further method of epiphysiodesis by placing two screws obliquely across the physis. The priniciple of this technique is based on applying compressive forces onto the physis [7].

The first temporary and possibly reversible hemiepiphysiodesis was described by Blount. His technique avoids physical damage by bridging the physis. This technique was described as early as 1949 for the first time and is performed by implanting 2 or 3 staples [8]. Increasing the number of staples correlates with a higher risk of damaging the physis. Therefore, Blount’s technique requires an accurate calculation of the remaining growth as well [7]. Due to the risk of damaging the physis, operations using this technique are consequently performed towards the end of growth and not during childhood [3].

Steven et al. described a “tension band principle” technique for temporary hemiepiphysiodesis by implanting an eight-Plate® (Orthofix, Lewisville, USA), which can be applied in younger patients as well [9]. Using this implant, a plate bridging the physis is used and fixed by two screws that are not fixed-angle. These plates lead to a temporary hemiepiphysiodesis with a good pressure distribution around the physis [3]. Different retrospective studies showed that eight-Plate® are as effective as staple hemiepiphysiodesis for guided growth in cases of angular deformity, even in younger patients [3, 10, 11].

Another implant, with a similar functional principle for guided growth, is the “FlexTack ^TM^” (Merete, Berlin, Germany), which in contrast to the eight-Plate® is a one-piece implant without mechanical slackness.

This biomechanical in-vitro study was initiated to compare the biomechanical characteristics of this new FlexTack^TM^ implant with the well-known eight-Plate® implant. Due to differences in design and implantation technique, it was hypothesised:

1. that the FlexTack^TM^ can bear larger forces than the eight-Plate® before implant failure is observed and
2. that the FlexTack^TM^ has a higher corrective potential as it develops momentum right after implantation, while the eight-Plate® has to experience angulation first to overcome initial mechanical slackness [11, 12].

## Materials and methods

**Fig. 1:**
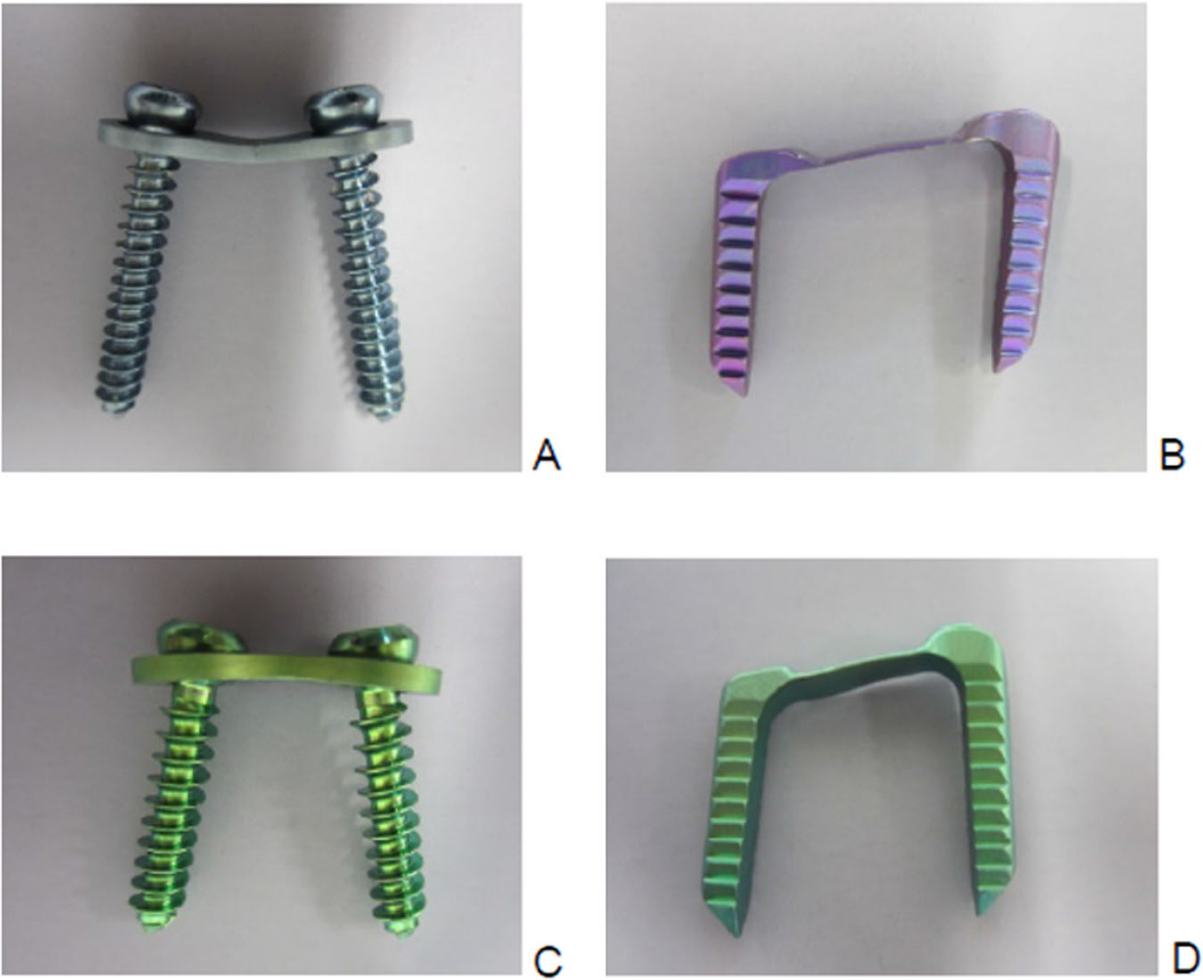
Shown are both tested implants. Smaller-sized implants of each type were used for anterior implantation, while the larger-sized implants were implanted laterally. **A** 8-Plate lateral (16 mm) The plate shown in this picture was photographed after explantation. Therefore, the plate is bent retro-convex; **B** FlexTack lateral (30 mm); **C** 8-Plate anterior (12 mm); **D** FlexTack anterior (25 mm)

In this study, eight-Plate® (plate and screws) and “FlexTack^TM^” (flexible staples; Fig. 1) were tested for biomechanical features. For implant testing 40 fresh frozen pig femora, harvested at animal’s age of 9–12 months were used. The existence of an open physis in the femora was verified by x-ray and subsequent macroscopic examination when removing attached soft tissue from the bones. The femora were stored in a vacuum plastic bag and frozen at −24 °C before being used for testing. 24 h before implantation of eight-Plate® or FlexTack^TM^, the femora samples were defrosted and kept at 6 °C. Before implantation, the cortical bone was cut at the height of the distal femoral physis. The opposite side of the femoral bone was opened as well to confirm the anatomical structure of the physis. The femoral shaft and the distal femoral condyle were fixed with polymethylmethacrylat (PMMA cement) (Technovit 3040, Heraeus Kulzer, Wehrheim, Germany). Samples were subsequently randomised into four groups á 10 samples each.

**Fig. 2:**
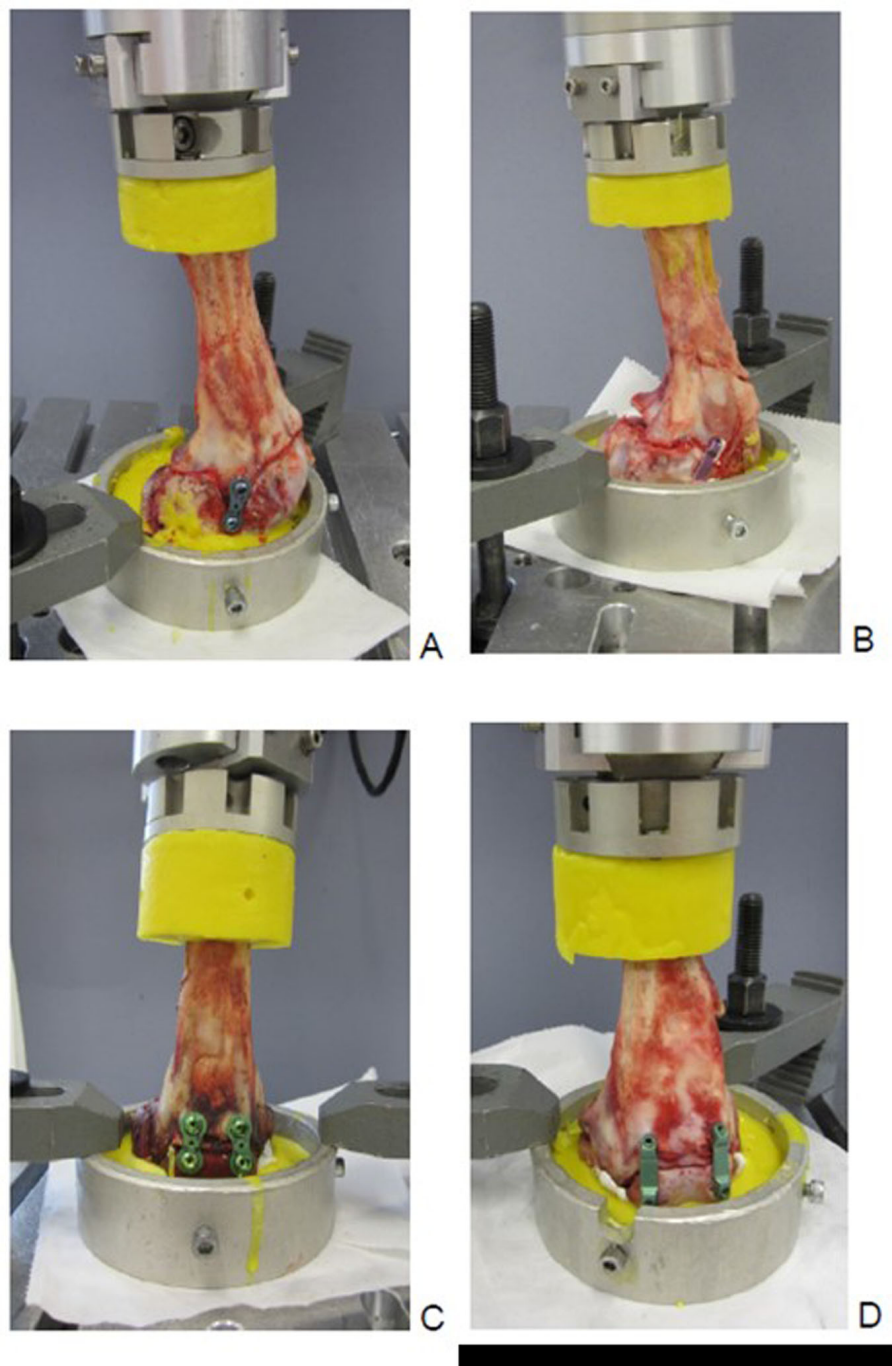
Shown are the four groups in the biomechanical testing machine. **A** Group 1: 1x Eight Plate lateral, **B** Group 2: 1x FlexTack lateral, **C** Group 3: 2x Eight Plate anterior and **D** Group 4: 2x FlexTack anterior

**Fig. 3:**
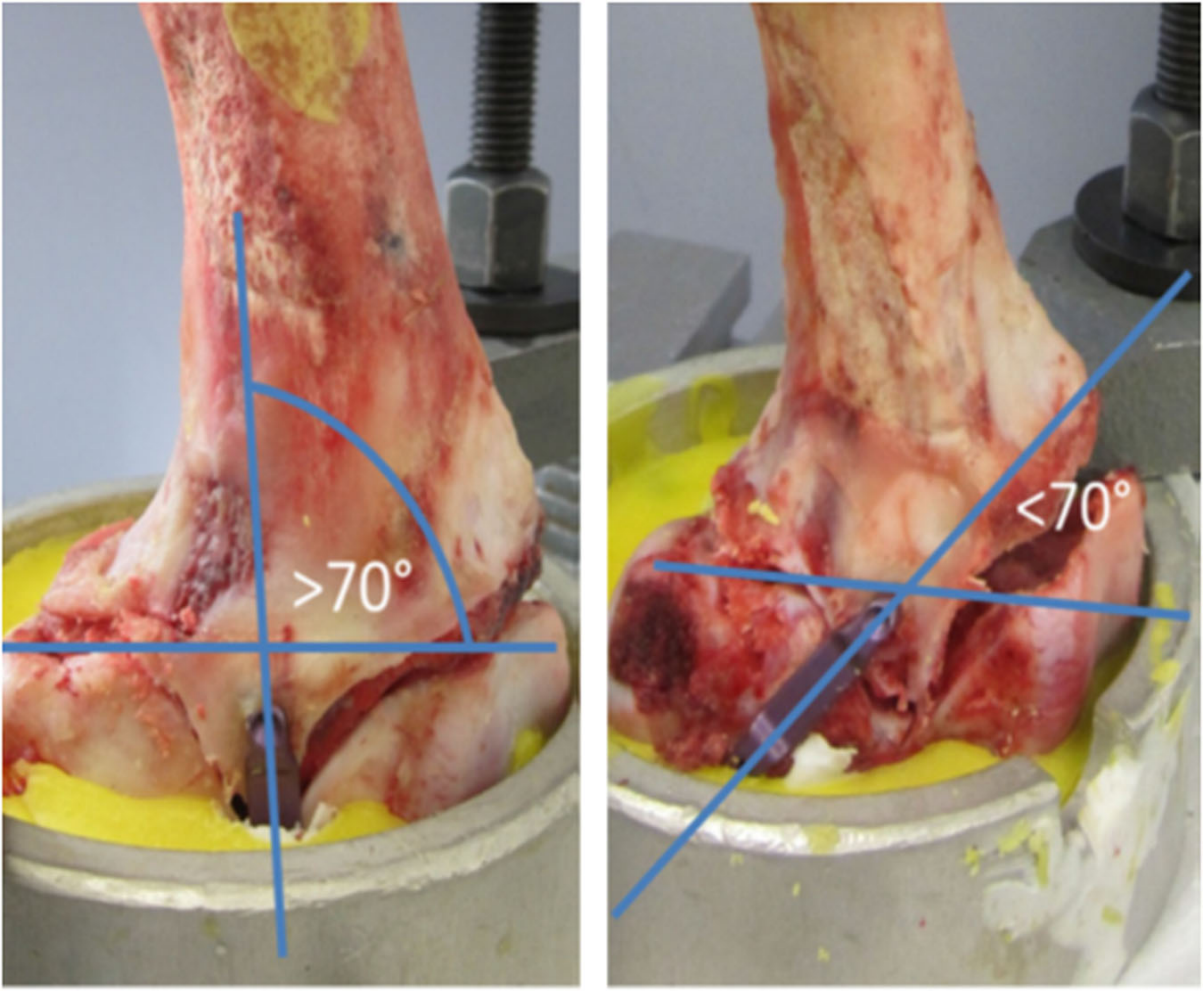
This figure demonstrates the difference between subgroups for lateral implantation of either implant type with more or less than 70° of angulation

Group 1 tested laterally implanted eight-Plate®, and Group 3 anteriorly implanted eight-Plate®. In analogy, groups 2 and 4 represented laterally and anteriorly implanted FlexTack^TM^. To test for the influence of implant positioning with regard to the femoral axis, groups 1 and 2 were divided in two subgroups each, including rectangular (>70°) and angulated (<70 °) implants (Figs. 2 and 3). In the FlexTack^TM^ group samples were distributed even. In the eight-Plate® group there was a slight mismatch with less rectangular (4) and more angulated (6) samples. This difference in distribution is explained by an angel of less than 70° in one case that was initially assigned to the rectangular group.

For biomechanical evaluation, prepared femora were fixed in a servo hydraulic material testing machine (858 Mini Bionix 2, MTS, MN USA). The experimental set up was constructed as an axial pull out test. Strain was carried out with a preload pressure of 20 N and a constant speed of 10 mm/min. The fixation in the material testing machine was performed by a flange coupling and a cardan joint. The forces exerted (N) and the axial opening of the physis (mm) were recorded with a frequency of 100 Hz. A broken implant or an emigration of the implant were considered as fixation failure.

**Fig. 4:**
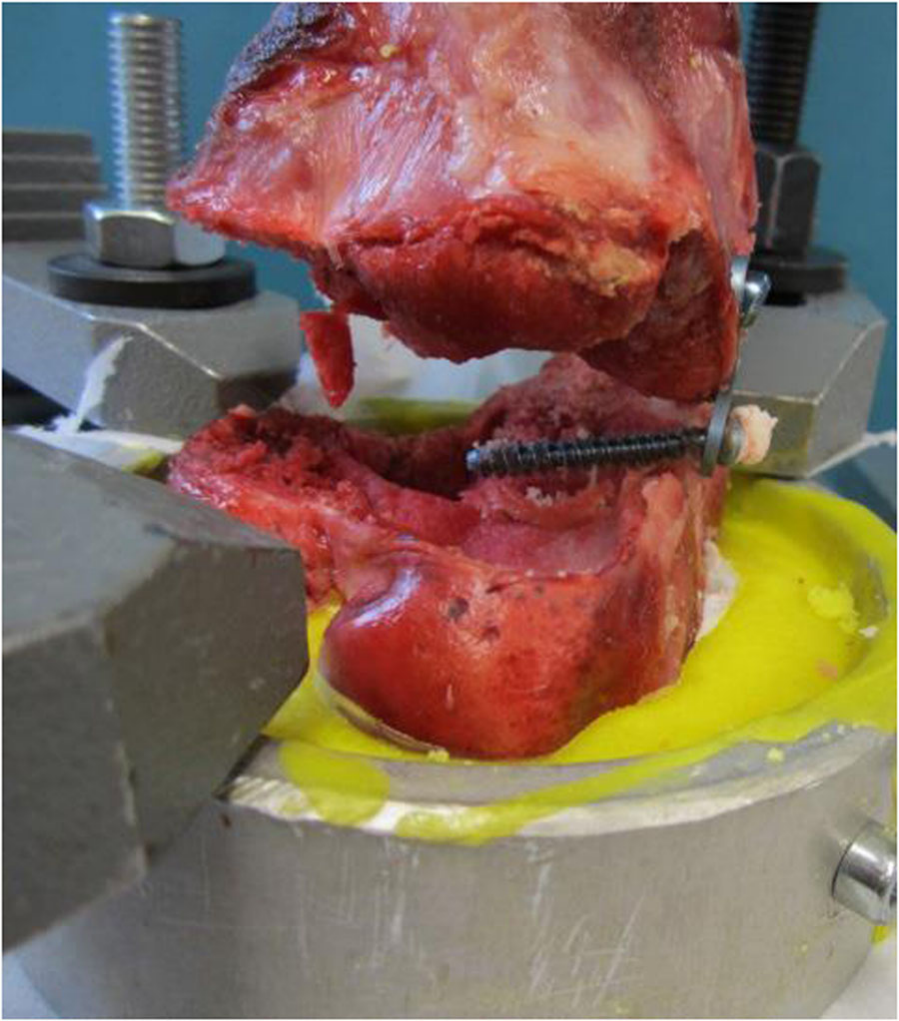
Demonstrated is the endpoint for “fixation failure”. Here, an 8-Plate is shown and the distal screw is broken out of the femoral bone stock

Final outcome parameters were axial distance until fixation failure (*l*_max_ in mm) and the maximum loads (*F*_max_ in N) applicable to the implant before failure. Forces required for axial distraction of the physis of 2 mm and 4 mm were measured (*F*_2mm_ and *F*_4mm_; Fig. 4).

The statistical analysis was carried out with SPSS 21.0 (IBM Corporation, Armonk, NY) software. Based on the Kolmogorov Smirnov test, the data was tested for normal distribution, followed by simple t-testing; *p* < 0.05 was considered significant.

## Results

### A. eight-Plate® lateral (Group 1) and FlexTack^TM^ lateral (Group 2)

**Fig. 5:**
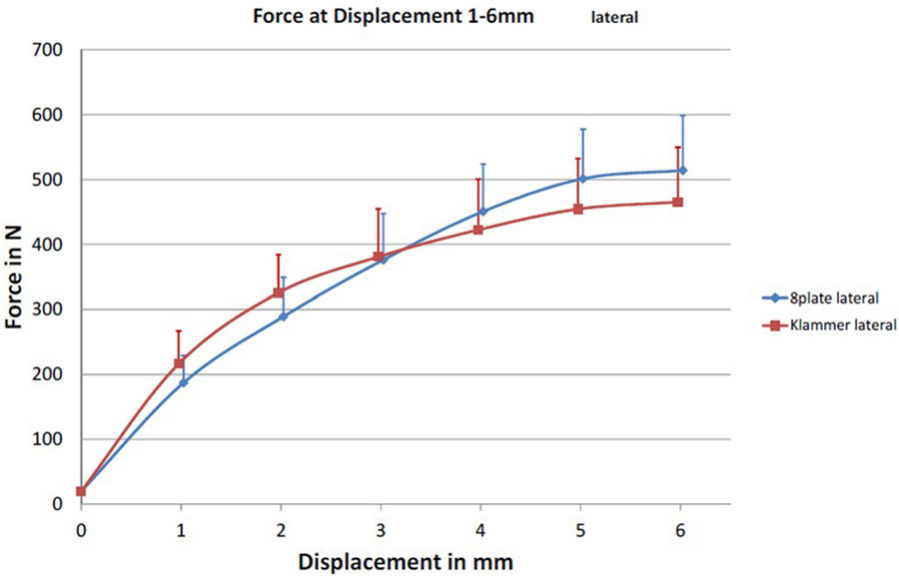
Axial displacement of the physis in mm and the corresponding exerted force in N for lateral implanted Eight Plates (blue and dots) and FlexTacks (red and squares)

There was no significant difference (*p* = 0.46) between the average *F*_max_ for the eight-Plate® lateral group (Group 1; 550 ± 157.5 N) and the FlexTack^TM^ lateral group (Group 2; 506.1 ± 91.3 N). *F*_2mm_ (Group 1: 289.2 ± 83.7 N vs. Group 2: 216.3 ± 40.6 N; *p* = 0.27) and *F*_4mm_ (Group 1: 450.5 ± 120.8 N vs. Group 2: 422.2 ± 52.9 N; *p* = 0.51) did not differ significantly between both groups. There were no significant differences in average forces at 1–6 mm displacement and for *l*_max_ (Group 1: 7.1 ± 2.3 mm vs. Group 2: 7.7 ± 2.7 mm; *p* = 0.65; Fig. 5*)*.

In Group 1, four plates were implanted angulated and six were implanted rectangular. There was no statistically significant difference between both types of angulation concerning *F*_max_ (*p* = 0.19) and *l*_max_ (*p* = 0.18). In Group 2, half of the FlexTack^TM^ were implanted straight and the remaining five were implanted rectangular. No significant differences concerning *F*_max_ (*p* = 0.49) or *l*_max_ (*p* = 0.09) were found.

In five cases of Group 1 the reason for the implant failure was a screw breakout with bone tissue and in the other five cases cut-out of the screws without bone tissue. In Group 2 only two implant failures were associated with the original bone tissue breakout.

### B. eight-Plate® anterior (Group 3) and FlexTack^TM^ anterior (Group 4)

**Fig. 6:**
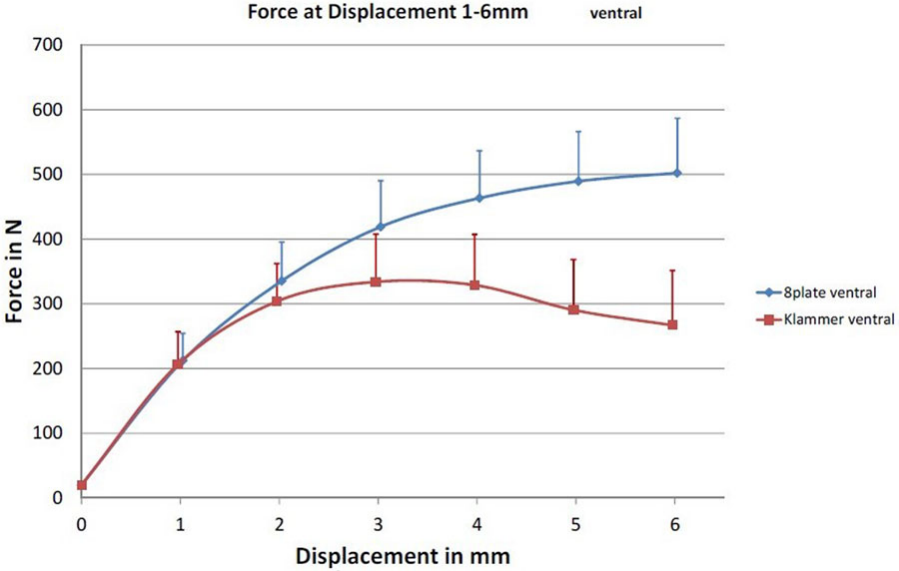
Axial displacement of the physis in mm and the corresponding exerted force in N for anterior implanted Eight Plates (blue and dots) and FlexTacks (red and squares)

In Group 3, average *F*_max_ was significantly higher (544.9 ± 87.4 N) than average *F*_max_ in Group 4 (371.5 ± 60.3 N; *p* < 0.001). *F*_4mm_ was significant higher for Group 3 than for Group 4 (Group 3: 463.3 ± 73.2 N vs. Group 4: 328.8 ± 78.3 N; *p* = 0.01). There was no significant difference for *F*_2mm_ between both groups (Group 3: 335.4 ± 59.9 N vs. Group 4: 303.6 ± 58.7 N; *p* = 0.25; Fig. 6*)*.

*l*_max_ was significantly larger for Group 3 compared to Group 4 (Group 3: 6.8 ± 1.8 mm vs. Group 4: 3.9 ± 1.2 mm; *p* = 0.001).

In nine of ten cases, the reason for implant failure in Group 3 was a screw breakout with a fracture apart from the screw (screw within bony tissue) and in one case a solitary breakout of the screw without adherent bone tissue.

In Group 4, three of ten cases showed a cut-out of the FlexTack^TM^ implant with a bone flake and seven cases a cut-out without adherent osseous flake.

## Discussion

Angular osseous deformities of the lower extremity during the growth period can be treated by different surgical techniques. In fact, there are permanent epiphysiodeses using the Phemister technique and temporary epiphysiodeses using Blount staples as traditional options to correct angular deformities. Both of these techniques require accurate calculation of the remaining growth as the primary requires destruction of the epiphyseal plate and the temporary epiphysiodesis according to Blount exposes an increased risk of epiphyseal plate injury.

An alternative surgical solution was introduced by eight-Plate® implants. The principle of this technique is tethering of the physeal periphery while enabling growth in the rest of the physis [13]. It is a well-described and established method to correct coronal plane deformities of the knee with minimal complications and has been tested in various studies [14, 15]. An implant, which was introduced more recently, is the FlexTack^TM^ implant, which follows a similar functional principle. The advantage of both techniques compared to the definitive epiphysiodesis is, that implants can be removed once the angular deformity has been corrected. So far, however, there are no biomechanical or clinical studies comparing both solutions.

This is the first study to compare biomechanical features of eight-Plate® and FlexTack^TM^ in a porcine in vitro model. Although both implants differ in design, there was no difference detected between both implant types regarding maximum loads (*F*_max_) and axial physis distance until fixation failure (*l*_max_) when implanted laterally. When implanted anteriorly, FlexTack^TM^ tended to fail at significantly lower maximum loads and showed significantly shorter axial physis distances until failure compared to eight-Plate®.

It remains questionable whether this difference between both implants affects clinical routine as most implants are brought in laterally/medially to correct varus/valgus deformities and only a minor percentage is implanted anteriorly for correction of flexion deformities of the knee (e.g. in neuromuscular disorders). This study was performed on porcine distal lateral and anterior femora and tested for osseous stability only. It did not respect soft tissue—implant interaction. In clinical routine, we made the experience that patients tend to complain more often about soft tissue irritation when treated by eight-Plate® than by FlexTack^TM^.

It is not exactly clear which forces are acting on the implants in-vivo as tendons, muscles and connective tissue may reduce distractive forces. It remains discussable if the implant behaviour of eight-Plate® changes in vivo after some weeks as its initial mechanical slackness between plate and screws will be turned into rigidity after a certain degree of angulation has been reached. In contrast, FlexTack^TM^ are primarily fixed-angle without mechanical slackness—which has the advantage of immediate correction potential. This fact may contribute to earlier implant failure in the present study as eight-Plate® yield a an initial greater distraction stability due to the mechanical slackness [16]. In the present study, two implants were implanted anteriorly and one only laterally, which was based on our clinical standard and experience.

Although there is initial mechanical slackness between screws and plate in eight-Plate® implants, screw threads yield a high initial stability within bone. Considering the implantation mechanics of FlexTack^TM^ staples it may be assumed—although staple’s legs are barbed—that they are initially less strong fixated within osseous tissue. However, the titanium alloy microstructured surface of the later allows for a rapid osteointegration, which results in a strong bone purchase after some weeks. In clinical practice, this fact requires special instruments (U-shaped chisels) for removal of deeply integrated FlexTack^TM^ staple legs, while eight-Plate® screws can be removed more easily. Thus, the present study cannot replicate implant-depend osteointegrative features.

The present study demonstrated that there are no biomechanical deficits when implanting the mentioned devices in either a rectangular or angular fashion.

Thus, clinical studies need to evaluate and directly compare both, eight-Plate® and FlexTack^TM^ with regard to clinical and radiological correction potential, adverse events, patient’s convenience and cost-benefit analysis.

## Conclusion

For lateral temporary epiphysiodesis there is no biomechanical difference between eight-Plate® and FlexTack^TM^ regarding the maximum loads bearable by the implants as well as the maximal distraction distance of implants until failure. In anterior temporary ephiphysiodesis, eight-Plate® demonstrated superior biomechanical features regarding the above mentioned parameters. At this stage of research, there is no clear preference towards one or the other implant and the choice remains within the individual preferences of the surgeon.
